# Triptonide Inhibits the Cervical Cancer Cell Growth via Downregulating the RTKs and Inactivating the Akt-mTOR Pathway

**DOI:** 10.1155/2022/8550817

**Published:** 2022-11-09

**Authors:** Li-na Zhou, Shi-qing Peng, Xue-Lian Chen, Xiao-ren Zhu, An-qi Jin, Yuan-yuan Liu, Li-xia Zhu, Ya-qun Zhu

**Affiliations:** ^1^Department of Radiotherapy & Oncology, The Second Affiliated Hospital of Soochow University, Institute of Radiation Oncology, Soochow University, 215004 Suzhou, China; ^2^Department of Radiotherapy and Oncology, Affiliated Kunshan Hospital of Jiangsu University, 215300 Kunshan, China; ^3^Department of Radiology, Affiliated Kunshan Hospital of Jiangsu University, Kunshan 215300, China; ^4^Clinical Research and Lab Center, Affiliated Kunshan Hospital of Jiangsu University, 215300 Kunshan, China; ^5^Department of Gynaecology and Obstetrics, Affiliated Kunshan Hospital of Jiangsu University, 215300 Kunshan, China

## Abstract

The high incidence and mortality of cervical cancer (CC) require an urgent need for exploring novel valuable therapeutics. Triptonide (TN) is a small molecule monomer extracted from the Chinese herb *Tripterygium wilfordii Hook*. Our results showed that TN, at only nanomolar concentrations, strongly inhibited growth, colony formation, proliferation, migration, and invasion of established and primary human cervical cancer cells. TN induced apoptosis and cell cycle arrest in cervical cancer cells. Moreover, cervical cancer cell *in vitro* migration and invasion were suppressed by TN. It was however noncytotoxic and proapoptotic to normal cervical epithelial cells and human skin fibroblast cells. Gene set enrichment analysis (GSEA) of RNA sequencing data of differentially expressed genes (DEGs) in TN-treated cervical cancer cells implied that DEGs were enriched in the receptor tyrosine kinase (RTK) signaling and PI3K-Akt-mTOR cascade. In cervical cancer cells, RTKs, including EGFR and PDGFR*α*, were significantly downregulated and Akt-mTOR activation was largely inhibited after TN treatment. *In vivo*, oral administration of TN significantly inhibited subcutaneous cervical cancer xenograft growth in nude mice. EGFR and PDGFR*α* downregulation as well as Akt-mTOR inactivation was detected in TN-treated HeLa xenograft tumor tissues. Thus, TN inhibits human cervical cancer cell growth *in vitro* and *in vivo*. Its anticervical cancer activity was associated with RTK downregulation and Akt-mTOR inactivation.

## 1. Introduction

Cervical cancer is the fourth most common cancer among women worldwide [1–3], and it is a leading cause of death in women in China, with around 98900 newly diagnosed cases and nearly 30500 new deaths in 2015 according to the National Central Cancer Registry of China (NCCRC) [4]. Persistent high-risk human papillomavirus (HPV) infection contributes to the carcinogenesis of cervical cancer [5–7]. Despite many advances in the prevention and screening methods of CC, the incidence of cervical cancer remains high worldwide; a large proportion of cervical cancer patients are diagnosed in advanced stages [8, 9]. Current treatments, including chemotherapy, radical surgery, and radiotherapy, show no major outstanding improvements on prognosis for patients with metastatic or recurrent diseases [10, 11]. In particular, platinum-based chemotherapy has been the main treatment of metastatic cervical cancer for years with no obvious improvement on the patients' survival [12]. Hence, the discovery of novel molecularly targeted agents is in urgent need for improving the therapeutic efficacy and restricting the harmful effects of cervical cancer [13–15].

The two main diterpene triepoxides Triptolide (TL) and Triptonide (TN) extracted from the Chinese herb *Tripterygium wilfordii Hook* have similar molecular structures, except that the C-14 position of TN is a carbonyl group while the C-14 position of TL is a hydroxyl group [16, 17]. TL is the main active component of *Tripterygium wilfordii*. Studies have shown that TL possesses immunosuppressive, anti-inflammatory, and antitumor effects [18–20], while clinically it is limited due to its poor solubility and severe hepatotoxicity and nephrotoxicity [21–23]. Triptonide (TN) has been utilized to treat rheumatoid arthritis for centuries [24]. TN has multiple biological functions including anti-inflammation, antifertility, and neuroprotective effects [25]. Recently, the hypotoxicity of TN with potent antitumor activity has been confirmed in different cancers [26–31]. Yet, the potential effect of TN on cervical cancer and the underlying molecular mechanisms have not been studied thus far.

Receptor tyrosine kinases (RTKs) are cell-surface growth-factor receptors with tyrosine kinase activity. Activation of RTKs plays a crucial role in a variety of cellular functions including proliferation, differentiation, and metabolism [32–34]. Dysregulation and sustained activation of several RTKs, including VEGFR, HER2/ErbB2, MET, EGFR, and PDGFR, as well as its downstream oncogenic cascades can lead to cervical cancer tumorigenesis, which are associated with poor prognosis and chemoresistance for cervical cancer [34–39]. In the neoplastic development and progression, activated RTKs cause activation of the downstream signaling PI3K/Akt/mTOR cascade, contributing to tumor progression, apoptosis resistance, and metastasis [36, 40–42]. This cascade is now an established and crucial therapeutic target of cervical cancer [43]. Here, we will investigate the potential effects of TN on human cervical cancer cell growth both *in vitro* and *in vivo*.

## 2. Materials and Methods

### 2.1. Ethics

The study was approved by the Ethics Review Board of Jiangsu University.

### 2.2. Chemicals and Reagents

Triptonide (TN, purity ≥ 98%) was purchased from Jiuzhi Chemicals (Shanghai, China). The detailed information about dissolution or dilution of TN was described in our previous study [30]. Cell culture reagents were supplied by Gibco (Vienna, Austria). The primary antibodies used were anti-cyclin D1 (#ab226977, Abcam), anti-cleaved poly (ADP-ribose) polymerase (PARP, E51, #ab203467, Abcam), anti-cleaved PARP (Y34, #ab237434, Abcam), anti-epidermal growth factor receptor (EGFR, sc-03, Santa Cruz), anti-platelet-derived growth factor receptor (PDGFR*α*) (sc-338, Santa Cruz), anti-phospho-mammalian target of rapamycin (*mTOR*, Ser 2448, #ab109268, Abcam), anti-phospho Akt (Ser 473) (#3787, Cell Signaling Technology), anti-phospho-S6 (Ser 235/236) (#2211 Cell Signaling Technology), anti-phospho-eukaryotic translation initiation factor 4E-binding protein 1 (4EBP1, Ser 65, #9451 Cell Signaling Technology), and anti-*β*-actin (#ab8227, Abcam). All chemicals were purchased from Sigma (St. Louis, MO).

### 2.3. Established Cells

The established human cervical cancer cell lines, including HeLa, C33a, and SiHa, human skin fibroblasts (HSF), and immortalized Ect1/E6E7 cervical epithelial cells were purchased from the Cell Bank of Shanghai Institute for Biological Science (Shanghai, China). Cells were cultured in high-glucose (4.5 g/L glucose) DMEM plus 10% FBS (Gibco, Vienna, Austria) and 100 U/mL penicillin-100 *μ*g/mL streptomycin (Gibco, Vienna, Austria) medium. Cells were then placed in an incubator with humidified atmosphere of 5% CO_2_ at 37°C. All cell lines were free of mycoplasma and microbial contamination examination. STR profiling was checked every three months to confirm the genotype.

### 2.4. Primary Human Cells

The fresh cervical cancer tissues or the paracancerous epithelial tissues were cut into small pieces and were digested by collagenase type I and dispase II. The digested human cells were washed, centrifuged, and incubated in complete medium with penicillin/streptomycin and DNase (500 U). Cell suspensions were thereafter filtered, centrifuged, and resuspended. Vascular cells, fibroblasts, and immune cells were removed. The primary cancer cells or cervical epithelial cells were cultivated in high-glucose DMEM/F-12 growth medium with 12% FBS, 0.25 *μ*g/mL hydrocortisone, 7.5 *μ*g/mL insulin, 25 *μ*g/mL epidermal growth factor (EGF), and 30 *μ*g/mL adenine. Here, the primary human cervical cancer cells (“priCCa-1,” “priCCa-2,” and “priCCa-3,” derived from three primary patients) and the primary human cervical epithelial cells (HCerEpC) were obtained. The protocols of using human cells were approved by the Ethics Board of Jiangsu University and were according to the principles of Helsinki declaration.

### 2.5. Reactive Oxygen Species (ROS) Detection

The intracellular ROS production was detected by a cellular ROS assay kit (#ab113851, Abcam). Briefly, cells were initially seeded into six-well plates for 24 h and treated with TN for the next 10 h; cells were then collected in the tubes and stained with medium containing 2′,7′-dichlorofluorescein diacetate (DCFH2-DA) dye for 30 min at 37°C. The fluorescent intensity was analyzed with a flow cytometer (BD Biosciences, CA).

### 2.6. Transcriptional Profiling (RNA Sequencing)

Transcriptome sequencing analysis was described in detail in our previous report [44], and differentially expressed genes (DEGs) were analyzed. Gene set enrichment analysis (GSEA) was used to assess its distribution trend in the phenotypic relevance sequencing gene list and determine its contribution to the phenotype. The relevancy to the phenotype was ranked by logFC value.

Other methods, including colony formation, cell counting kit-8 (CCK-8) assaying of cell viability, nuclear 5′-ethynyl-2′-deoxyuridine (EdU)/Hoechst-33342 (or DAPI) staining of cell proliferation, “Transwell” and “Matrigel Transwell” assays, terminal deoxynucleotidyl transferase dUTP nick end labeling (TUNEL)/DAPI nuclei staining, *propidium iodide*- (*PI*-) Annexin V flow cytometry, caspase-3 activity assay, cell cycle progression by PI-FACS, *in vitro* wound-healing assay, mitochondrial JC-1 (the mitochondrial membrane potential probe) staining of mitochondrial depolarization, and Western blotting, were described in detail in our previous studies [44–48].

### 2.7. Tumor Xenograft Experiments

Briefly, HeLa cells (1 × 10^7^ cells per mouse, in 50 *μ*L DMEM plus 50 *μ*L Matrigel) were inoculated into the five-week-old female BALB/c nude mice (half male half female, 18.5-19.0 g). Within three weeks, the tumor volume reached around 100 mm^3^, and the mice were intragastrically administrated daily with either Triptonide (TN, 10 mg/kg daily) or saline (“Ctr”) for 21 consecutive days. Tumor volumes were measured weekly and calculated by *π*/6 × larger diameter × (smaller diameter)^2^. Estimated daily tumor growth (in mm^3^/day) was calculated [49]. Xenograft slide immunohistochemistry (IHC) protocols were reported previously [48]. Animal experiments were approved by the Institutional Animal Care and Use Committee (IACUC) and the Ethics Review Board of Jiangsu University.

### 2.8. Tissue TUNEL Staining

Briefly, TUNEL staining was performed on 4% paraformaldehyde-fixed, paraffin-embedded tissue sections. After dewaxing, the tissue slices were covered with Protease K for 30 min at 37°C and then washed with PBS twice (2 min per time). Equilibration buffer was used to cover the tissue slices. After 30 min, FITC-dUTP labeling buffer was used to cover the slices for one hour at 37°C, followed by washing with PBS twice. Next, slices were stained with DAPI (#721621, GEPbio) and visualized under an Olympus FSX100 microscope (Olympus, Tokyo, Japan).

### 2.9. Statistical Analysis

*In vitro* experiments were repeated three to five times. Data in the present study were always normally distributed and were presented as mean ± standard deviation (SD). Statistical analyses were performed under the SPSS 23.0 software (SPSS Co., Chicago, IL). Unpaired student's *T*-test was utilized when comparing between two groups. One-way ANOVA plus the Scheffe and Tukey test was carried out for comparison of three and more groups. *P* values of <0.05 were considered as statistically significant. IC-50 (the concentration resulting in 50% CCK-8 viability reduction) was calculated by nonlinear regression analysis using GraphPad Prism 5.01.

## 3. Results

### 3.1. Triptonide Exerts Potent Cytotoxicity to Human Cervical Cancer Cells

**Figure 1:**
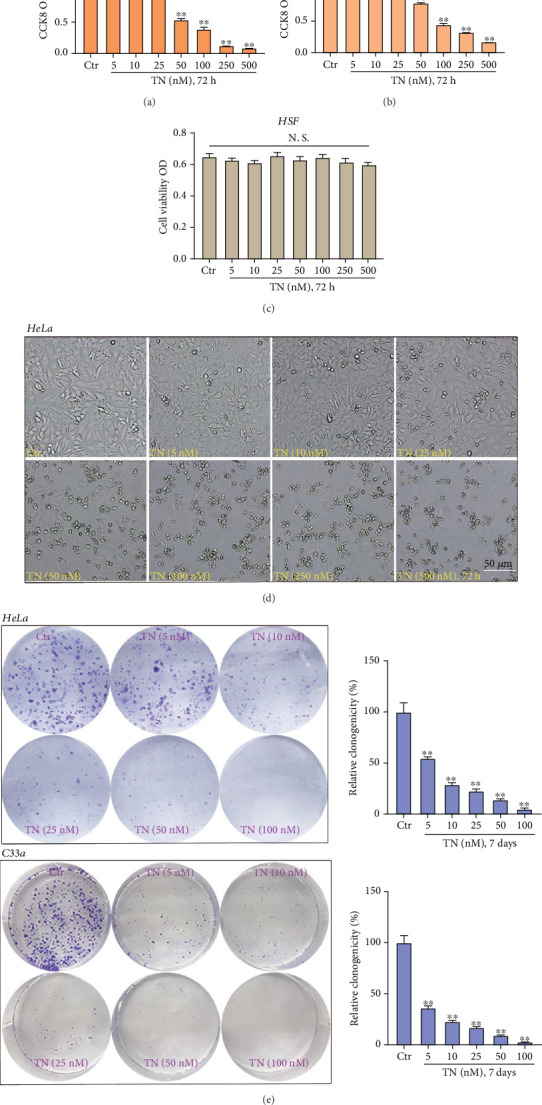
Triptonide exerts potent cytotoxicity to human cervical cancer cells. Established human cervical cancer cell lines (HeLa and C33a) or the human skin fibroblasts (HSF) were treated with vehicle control (“Ctr”) or the designated concentrations (5–500 nM) of Triptonide (TN); cells were further cultured in conditional (TN-containing) medium for applied time periods and were subjected to (a–c) CCK-8 assay of cell viability and (e) colony formation assay. The representative HeLa cell morphology images were presented (d). Data were represented as mean ± SD. For each assay, at least five independent experiments were repeated. “Ctr” stands for the vehicle control (0.1% DMSO). ⁣^∗^*P* < 0.05 vs. “Ctr.” Scale bar = 50 *μ*m.

The reduction of CCK-8 optical density (OD) was utilized to indicate viability reduction and cytotoxicity. As demonstrated, TN treatment dose-dependently inhibited the viability of HeLa cells and C33a cells (Figures 1(a) and 1(b)). Cell viability reduction was significant after 5 nM of TN treatment (72 h) in HeLa cells and C33a cells (Figures 1(a) and 1(b)). The IC-50 of TN, or the concentration resulting in 50% CCK-8 viability reduction, was between 20 and 50 nM (Figures 1(a) and 1(b)). Remarkably, the same treatment of TN (5-500 nM) failed to exert significant cytotoxicity to normal HSF (human skin fibroblasts), as the CCK-8 OD was not significantly decreased (Figure 1(c)). The representative HeLa cell morphology images (Figure 1(d)) further supported the cytotoxicity of TN. Moreover, the colony-forming ability of HeLa cells and C33a cells was robustly decreased following treatment of TN (5–100 nM) (Figure 1(e)). These results supported the selective and robust cytotoxicity of TN against cervical cancer cells.

### 3.2. Triptonide Inhibits Cervical Cancer Cell Proliferation and Cell Cycle Progression

**Figure 2:**
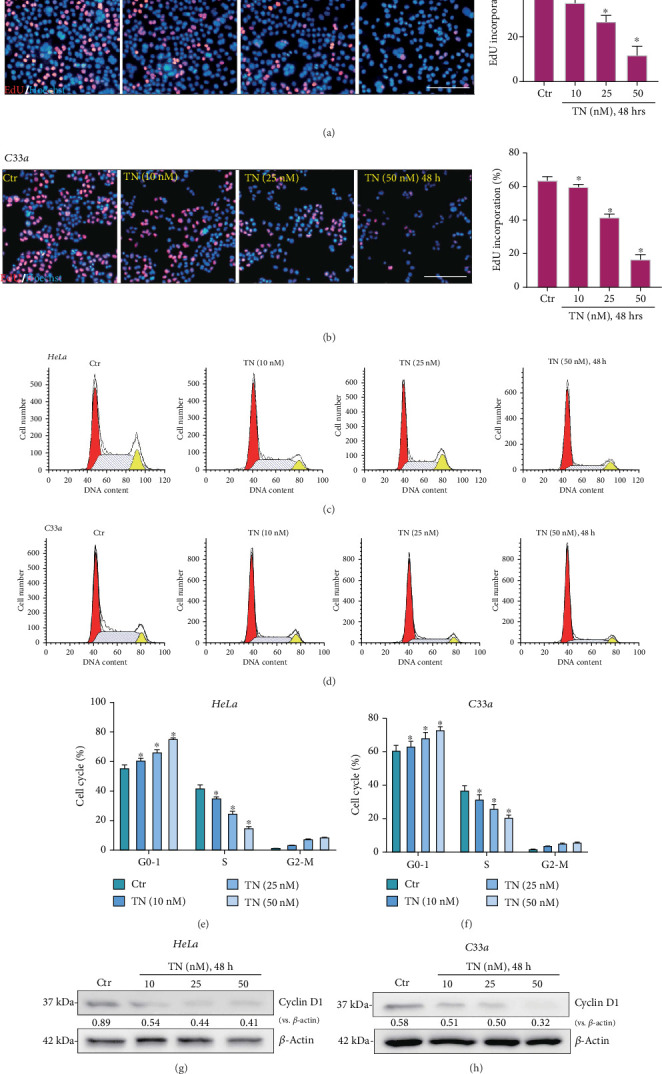
Triptonide inhibits cervical cancer cell proliferation and cell cycle progression. Human cervical cancer cells (HeLa and C33a) were treated with designated concentrations (10-50 nM) of Triptonide (“TN”); cells were then cultured in conditional (TN-containing) medium for indicated time, (a, b) cell proliferation (EdU incorporation staining assay) and (c–f) cell cycle progression (PI-FACS assays) were performed, and (g, h) expression of listed proteins in the total cell lysates was tested, with results quantified. Data were presented as mean ± SD. For each assay, at least five independent experiments were repeated. “Ctr” stands for the vehicle control (0.1% DMSO). ⁣^∗^*P* < 0.05 vs. “Ctr.” Scale bar = 100 *μ*m.

Furthermore, the potential effect of TN on human cervical cancer cell proliferation was tested. HeLa cells and C33a cells were treated with TN (10-50 nM). Performing the nuclear EdU staining assay, we found that TN (48 h) dose-dependently decreased EdU-positive nuclei ratio (EdU/Hoechst-33342 × 100%) in HeLa cells and C33a cells (Figures 2(a) and 2(b)). Cell cycle progression is critical for cancer cell proliferation [50]. In TN-treated cells, the percentages of G0/1-phase and G2/M-phase cells were increased (Figures 2(c) and 2(d)), while the percentage of S-phase cells was decreased (Figures 2(c) and 2(d)). Statistical analysis of cell cycle by integrating five replicated PI-FACS results showed that TN induced G1-S cell cycle arrest, and its effect was dose dependent (Figures 2(e) and 2(f)). Notably, expression of a key cell cycle-associated protein, cyclin D1, was significantly downregulated in TN-treated cervical cancer cells (Figures 2(g) and 2(h)), which might be a key mechanism of TN-induced cell cycle arrest and proliferation inhibition [51]. Collectively, TN inhibited human cervical cancer cell proliferation and cell cycle progression.

### 3.3. Triptonide Induces Apoptosis in Human Cervical Cancer Cells

**Figure 3:**
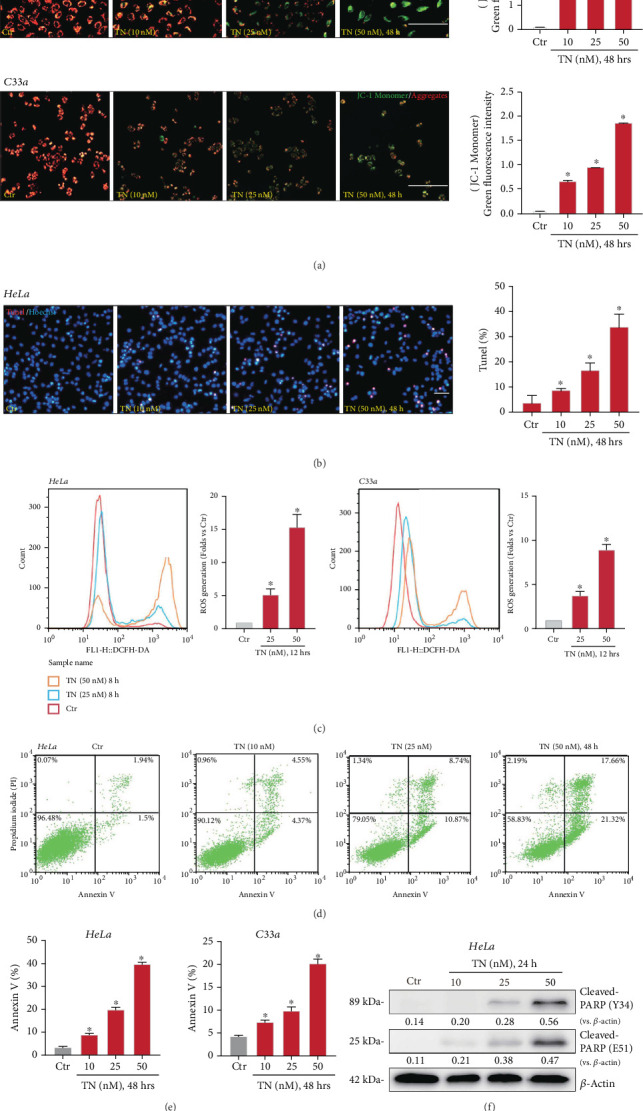
Triptonide induces apoptosis activation in human cervical cancer cells. Human cervical cancer cells (HeLa and C33a) were treated with designated concentrations (10–50 nM) of Triptonide (“TN”); cells were then cultured in conditional (TN-containing) medium for indicated time. Mitochondrial depolarization and reactive oxygen species (ROS) production were tested by (a) JC-1 staining and (c) DCFH-DA staining assays, respectively. Cell apoptosis was tested by (b) nuclear TUNEL staining and (d, e) Annexin V-FITC/PI flow cytometry. Expression of listed proteins was shown (f). Data were presented as mean ± SD. For each assay, at least five independent experiments were repeated. “Ctr” stands for the vehicle control (0.1% DMSO). ⁣^∗^*P* < 0.05 vs. “Ctr.” Scale bar = 50 *μ*m.

Since TN induced viability reduction, growth arrest, and proliferation inhibition in established human cervical cancer cells, we next examined its activity on cell apoptosis. In TN-treated HeLa cells and C33a cells, increased JC-1 green monomers were formed, indicating mitochondrial membrane potential depolarization (Figure 3(a)). In a dose-dependent manner, TN significantly increased the TUNEL-positive nuclei ratio (Figure 3(b)), supporting apoptosis activation. Significant ROS production was detected in TN-treated cervical cancer cells, evidenced by the DCFH-DA intensity increasing (Figure 3(c)). Annexin V-FITC FACS assay results found that TN treatment (10–50 nM) dose-dependently increased the Annexin V-positive staining cervical cancer cells (Figures 3(d) and 3(e)). In addition, TN treatment induced cleavages of poly-ADP-ribose polymerase (PARP) in the cervical cancer cells (Figure 3(f)). Therefore, TN efficiently induced apoptosis in cervical cancer cells.

### 3.4. Triptonide Inhibits Human Cervical Cancer Cell Migration and Invasion *In Vitro*

**Figure 4:**
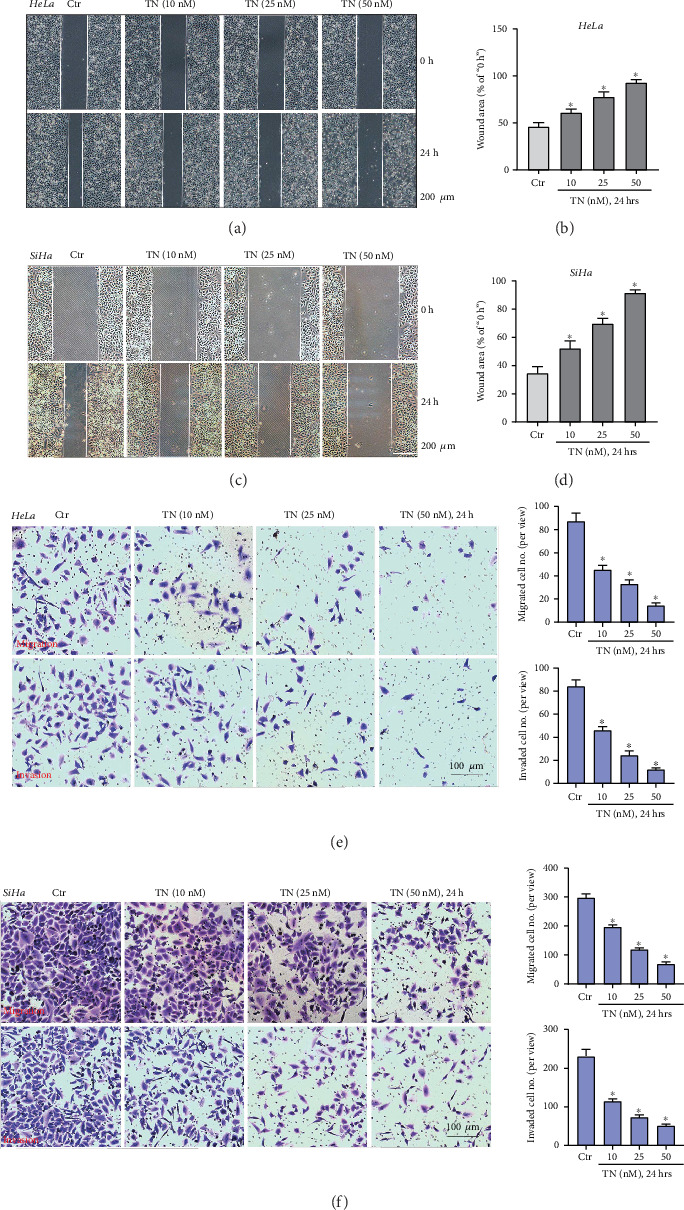
Triptonide inhibits human cervical cancer cell migration and invasion *in vitro.* Human cervical cancer cells (HeLa and SiHa) were treated with designated concentrations (10–50 nM) of Triptonide (“TN”); cell migration/invasion was evaluated by (a–d) “starch wound-healing assay”, (e) Transwell migration, and (f) Matrigel Transwell invasion assays. Data were presented as mean ± SD. For each assay, at least five independent experiments were repeated. “Ctr” stands for the vehicle control (0.1% DMSO). ⁣^∗^*P* < 0.05 vs. “Ctr”.

Then, we evaluated the potential effect of TN on cervical cancer cell motility. Wound-healing assay results revealed that TN (10–50 nM) dose-dependently inhibited wound closure in HeLa cells and SiHa cells (Figures 4(a)–4(d)). Moreover, TN markedly impeded HeLa and SiHa cell *in vitro* migration and invasion (Figures 4(e) and 4(f)), tested separately by “Transwell” and “Matrigel Transwell” assays.

### 3.5. TN Exerts Significant Anticancer Activity in Primary Cervical Cancer Cells

**Figure 5:**
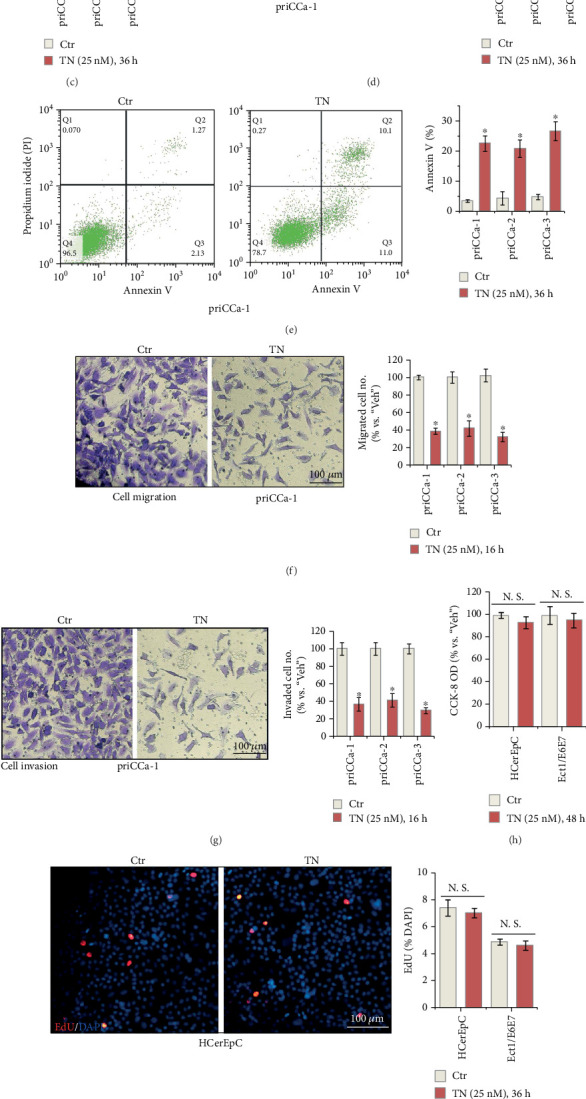
TN exerts significant anticancer activity in primary cervical cancer cells. (a–g) The primary human cervical cancer cells, priCCa-1, priCCa-2, and priCCa-3, (h–j) the primary human cervical epithelial cells (HCerEpC), or (h–j) the established Ect1/E6E7 epithelial cells were treated with Triptonide (“TN,” at 25 nM) and cultivated for indicated time periods; (a, h) cell viability and (b, i) proliferation, (c–e, j) apoptosis, and (f) cell migration and (g) invasion were examined by the corresponding assays. Data were presented as mean ± SD (*n* = 5). “Veh” stands for the vehicle control. ⁣^∗^*P* < 0.05 vs. “Veh” treatment. “N.S.” stands for the nonstatistical difference (*P* > 0.05). The experiments were repeated five times with similar results obtained. Scale bar = 100 *μ*m.

We also examined the potential effect of TN in primary cancer cells. Primary human cervical cancer cells that were derived from three different primary patients, namely, priCCa-1, priCCa-2, and priCCa-3, were cultured and treated with TN (25 nM). As demonstrated, TN treatment robustly decreased cell viability (CCK-8 OD reduction, Figure 5(a)) and proliferation (EdU-positive nuclei ratio decreasing, Figure 5(b)) in all three primary cervical cancer cells. Moreover, significant apoptosis activation was detected in TN-treated primary cancer cells. The caspase-3 activity (Figure 5(c)), TUNEL-positive nuclei percentage (Figure 5(d)), and Annexin V-positive staining (Figure 5(e)) were all significantly enhanced in TN-treated primary human cervical cancer cells. Supported by the results from “Transwell” and “Matrigel Transwell” assays, we showed that TN largely suppressed *in vitro* migration (Figure 5(e)) and invasion (Figure 5(f)) of priCCa-1/2/3 cells.

The potential effect of TN on noncancerous cervical epithelial cells was studied. The primary human cervical epithelial cells (HCerEpC) and the established Ect1/E6E7 cervical epithelial cells were treated with TN at the same concentration (25 nM). As demonstrated, TN failed to inhibit viability (CCK-8 OD, Figure 5(g)) and proliferation (by measuring EdU-positive nuclei ratio, Figure 5(h)) in HCerEpC and Ect1/E6E7 cells. Neither did it induce significant apoptosis (Figure 5(i)) in the epithelial cells. These results further supported a cancer cell-specific effect by TN.

### 3.6. Triptonide Induces Multiple Receptor Tyrosine Kinase Degradation and Suppresses PI3K/Akt/mTOR Signaling in Cervical Cancer Cells

**Figure 6:**
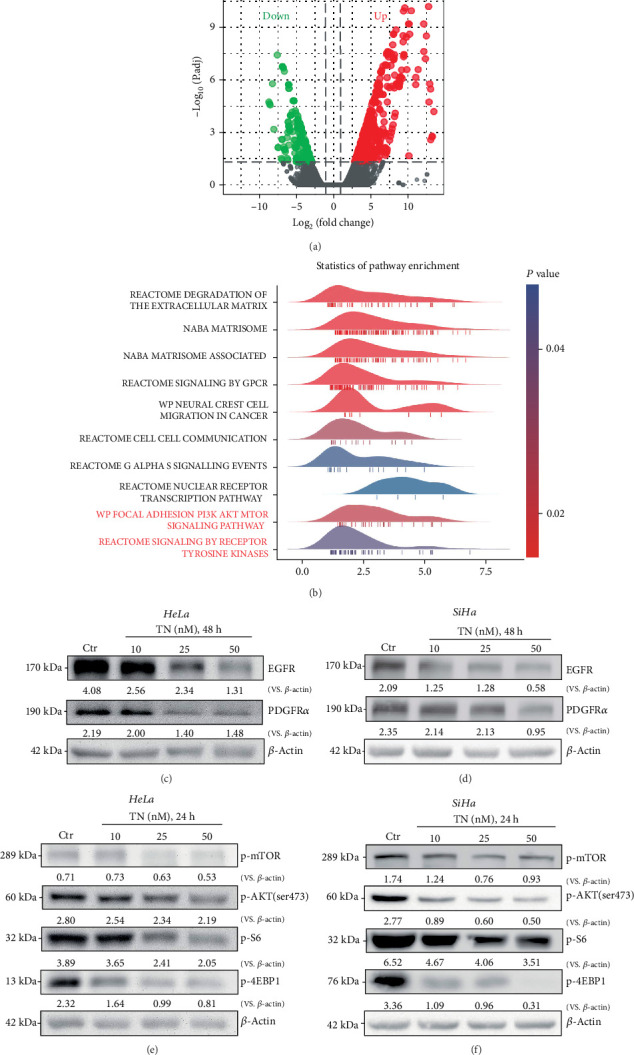
Triptonide induces RTK degradation and suppresses PI3K/Akt/mTOR signaling in cervical cancer cells. Volcano plots show the representative DEGs (both upregulated and downregulated genes) in TN-treated (50 nM) SiHa cells (a). Signaling pathway analyses of DEGs performed with GSEA are shown (b). Human cervical cancer cells (HeLa and SiHa) were treated with designated concentrations (10–50 nM) of Triptonide (“TN”); cells were then cultured in conditional (TN-containing) medium for indicated time periods. Expression of related RTKs (EGFR and PDGFR*α*) was examined (c, d). Expression of indicated PI3K/Akt/mTOR pathway proteins was examined as well (e, f). In this figure, experiments were repeated five times independently, and similar results were obtained. “Ctr” stands for the vehicle control (0.1% DMSO).

Next, we studied the possible underlying signaling mechanisms of TN-induced inhibition on cervical cancer cells. RNA-seq (high-throughput transcriptional profiling) was performed to analyze differentially expressed genes (DEGs) in TN-treated (50 nM) SiHa cells. The upregulated and downregulated DEGs in TN-treated cells were shown by plotting the volcano map (Figure 6(a)). Gene set enrichment analysis (GSEA) was used to identify the enriched pathways. Results showed that DEGs in TN-treated cells are enriched in the regulation of multiple signaling pathways (Figure 6(b)). Among them, the receptor tyrosine kinases (RTKs) and phosphatidylinositol-3-kinase- (PI3K-) Akt-mTOR cascades are significant (*P* < 0.05, “red font,” Figure 6(b)).

Confirming the RNA-seq results, we found that treatment with TN (10-50 nM, 48 h) in HeLa and SiHa cells resulted in robust downregulation of EGFR and PDGFR*α* (Figures 6(c) and 6(d)). Moreover, phosphorylation of mTOR, Akt, S6, and 4EBP1 was potently decreased in TN-treated (10-50 nM, 24 h) HeLa and SiHa cells (Figures 6(e) and 6(f)). Therefore, TN indeed induced degradation of EGFR and PDGFR*α* and suppressed PI3K/Akt/mTOR cascade in cervical cancer cells.

### 3.7. Triptonide Administration Inhibits Cervical Cancer Xenograft Tumor Growth in Mice

**Figure 7:**
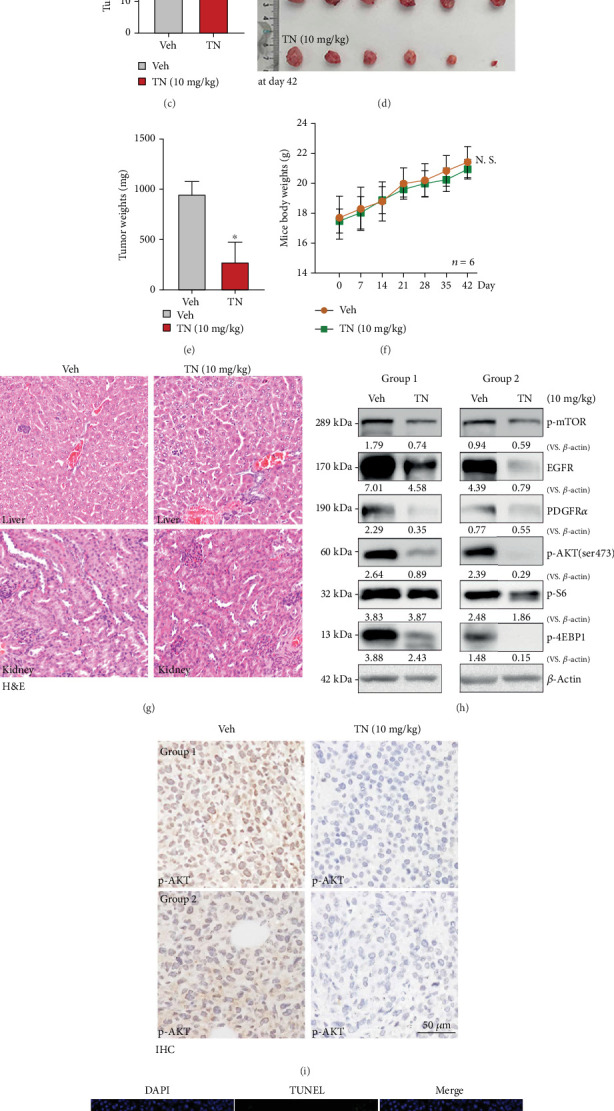
Triptonide oral administration inhibits cervical cancer xenograft tumor growth in mice. HeLa cells were subcutaneously injected to the right flanks of the nude mice (1 × 10^7^ cells per mouse) to establish xenograft tumors; TN (daily, 10 mg/kg mouse body weight, gavage, for 21 consecutive days) or saline control (“Veh”) treatment was started when tumor volume reached 100 mm^3^ (“day 0”). The images of tumor-bearing nude mice and the xenograft tumors at day 42 are shown (a, d, e). The (b) weekly tumor volumes and (f) mouse body weights were recorded. Estimated daily tumor growth (mm^3^ per day) was calculated (c). At day 42, xenograft tumors were isolated and the representative H&E images for liver and kidney tissues in both groups of mice are shown (g). Expression of listed proteins in the fresh xenograft tissues was tested (h). The representative IHC images of phosphorylated AKT (ser473) in xenograft slides are presented (i). Tissue slides were also subject to the immunofluorescence staining of TUNEL and DAPI (j). The number of mice per group, *n* = 6. ⁣^∗^*P* < 0.05 vs. saline-treated control mice (“Veh”). Scale bar = 50 *μ*m.

To study the potential *in vivo* anticervical cancer activity by TN, HeLa cells were injected into nude female mice subcutaneously, and the xenograft models were established (Figure 7(a)). When the tumor volume reached about 100 mm^3^ in total volume, mice were administrated with either TN daily (10 mg/kg, gavage, for 21 days, saline for control tumors) or the vehicle control. Tumor volumes were monitored every seven days following TN/vehicle injection. Tumor growth curve results confirmed that TN administration potently inhibited HeLa xenograft tumor growth in nude mice (Figure 7(b)). Compared with the control group, estimated daily tumor growth (in mm^3^/day) was also significantly lower in TN-treated mice (Figure 7(c)). The mice were humanely euthanized six weeks (day 42) following the first gavage of TN, tumor tissues were isolated, and tumor volumes and weights were measured. Xenograft tumors in the TN group were robustly smaller than those in the vehicle control mice (Figures 7(d) and 7(e)). Notably, mouse body weights were not significantly different between the two groups (Figure 7(f)). We also failed to observe obvious toxicities or complications in the TN-treated mice, which was further supported by immunohistochemistry assay of the liver and kidney in TN-treated mice (Figure 7(g)). Thus, the mice were well tolerated to the TN administration regimen, as reported in the previous study [30].

Fresh xenograft tissue lysates were analyzed, and we found that expression of EGFR and PDGFR*α* was significantly downregulated in TN-treated xenografts and phosphorylation of mTOR, Akt, S6, and 4EBP1 was decreased (Figure 7(h)). Moreover, the immunohistochemistry (IHC) staining results further supported that TN administration inhibited Akt phosphorylation in HeLa xenograft slides (Figure 7(i)). The xenograft slide immunofluorescence images confirmed that TUNEL-positive nuclei ratio was robustly increased in TN-treated HeLa xenografts, supporting apoptosis activation (Figure 7(j)). Together, TN inhibited RTK-PI3K/Akt/mTOR signaling and hindered HeLa xenograft growth in nude mice.

## 4. Discussion

Cervical cancer is a major cause of mortality among women worldwide despite the current frontline treatments, and there is an unmet need for patients with locally advanced cervical cancer [12, 52]. The clinical development of new effective targeted drugs for metastatic cervical cancer is extremely urgent [53].

Molecularly targeted therapies have become a promising option for cervical cancer [53]. Emerging studies show that Triptolide (TL) and Triptonide (TN), the two major active components of the *Tripterygium wilfordii Hook*, exhibit potent anticancer and anti-inflammatory activities [54]. TL inhibited cervical cancer by inactivating Akt/mTOR signaling and by inducing p53 and caspase-dependent cell death [55–57]. Yet its application is limited due to its poor solubility and bioavailability as well as potential toxicity [20].

Emerging studies have revealed the robust anticancer activity of TN in different human malignancies [26–31, 58, 59], including gastric tumor, osteosarcoma, nasopharyngeal carcinoma, melanoma, pancreatic cancer, and lymphoma. Zhang et al. showed that TN induced significant inhibition of cell proliferation and colony formation of lung cancer cells at low concentrations (5-10 nM), while simultaneously suppressing sphere-forming activity [26]. Here, we found that TN robustly inhibited human cervical cancer cell survival, colony formation, and proliferation. The anticervical cancer cell activity of TN was potent with an IC-50 lower than 50 nM. TN induced G1-S cell cycle arrest and apoptosis in cervical cancer cells. Significantly, TN exerted robust anticancer activity in multiple primary cervical cancer cells, while being noncytotoxic in cervical cancer epithelial cells. It has been demonstrated that TN potently inhibited melanoma cell and gastric cancer cell growth and metastasis via induction of SAV1 (salvador family WW domain-containing protein 1)/LATS1 (large tumor suppressor kinase 1) expression and activation of Hippo pathway [31] or via inhibition of the oncogenic Notch1 and NF-*κ*B signaling pathways [28]. Patients with cervical cancer were often diagnosed at advanced stages, with lymph node metastasis [60, 61]. Cell migration and invasion are essential for its progression [62, 63]. Here, we showed that TN potently suppressed human cervical cancer cell migration and invasion. Dong et al. reported that TN suppressed prostate cancer growth through inhibition of the mTOR cascade [27].

Receptor tyrosine kinases (RTKs) and PI3K-Akt-mTOR signaling play essential roles in multiple adverse biological behaviors including tumorigenesis and progression and are associated with poor prognosis and therapeutic resistance for several types of malignancy including cervical cancer [43, 64–66]. Liu et al. reported that overexpressed CD155 promoted cervical cancer progression by activating Akt-mTOR and NF-*κ*B cascade [67]. Cyanidin-3-O-glucoside plus cisplatin cotreatment or baicalein single treatment prevented PI3K-AKT-mTOR pathway activation and inhibited cervical cancer cell proliferation [68, 69]. Importantly, activation of the PI3K-AKT-mTOR signaling pathway by the human papillomavirus (HPV) oncogenes E6/E7/E5 plays a pivotal role in tumor initiation and progression [43].

GSEA of the RNA-seq data in the present study revealed that a total of 2458 genes were dysregulated in TN-treated cervical cancer cells, of which the RTKs and PI3K-Akt-mTOR cascades were significantly downregulated. Specifically, in TN-treated Hela and SiHa cells, EGFR and PDGFR*α* were significantly downregulated and Akt-mTOR signaling was largely inhibited. Furthermore, EGFR and PDGFR*α* downregulation and Akt-mTOR inactivation were detected in TN-treated HeLa xenografts. These results suggest that TN-induced inhibition of cervical cancer cell growth is associated with RTK (EGFR and PDGFR*α*) downregulation and PI3K-Akt-mTOR inactivation.

It has been reported that dysregulation of various oncogenic proteins, including E6/E7/E5 overexpression, could induce Akt-mTOR signaling hyperactivation to promote cervical cancer progression [70, 71]; it would be interesting to further explore the potential effect of TN on these oncogenic proteins. Further studies will also be needed to explore the underlying signaling mechanisms of RTK degradation and Akt-mTOR inactivation by TN.

## 5. Conclusion

Collectively, TN potently inhibits human cervical cancer cell growth *in vitro* and *in vivo*.

## Data Availability

All data are available upon request.

## Supplementary Materials

Supplementary Materials Figure S1: TN induced cervical cancer cell oxidative stress in a dose-dependent manner. Detection of mitochondrial superoxide (red) production by the MitoSOX staining assay in HeLa (A) and SiHa (B) cells. Scale bar = 100 *μ*m. *n* = 3 per group.
