# Supplementary material for: Triptonide Inhibits the Cervical Cancer Cell Growth via Downregulating the RTKs and Inactivating the Akt-mTOR Pathway
Source: Oxid Med Cell Longev. 2022 Nov 9;2022:8550817. doi: 10.1155/2022/8550817 (PMC11401660; doi:10.1155/2022/8550817)
Supplement: Supplementary Materials — Figure S1: TN induced cervical cancer cell oxidative stress in a dose-dependent manner. Detection of mitochondrial superoxide (red) production by the MitoSOX staining assay in HeLa (A) and SiHa (B) cells. Scale bar = 100 μm. n = 3 per group. [file 8550817.f1.pdf]

Figure S1

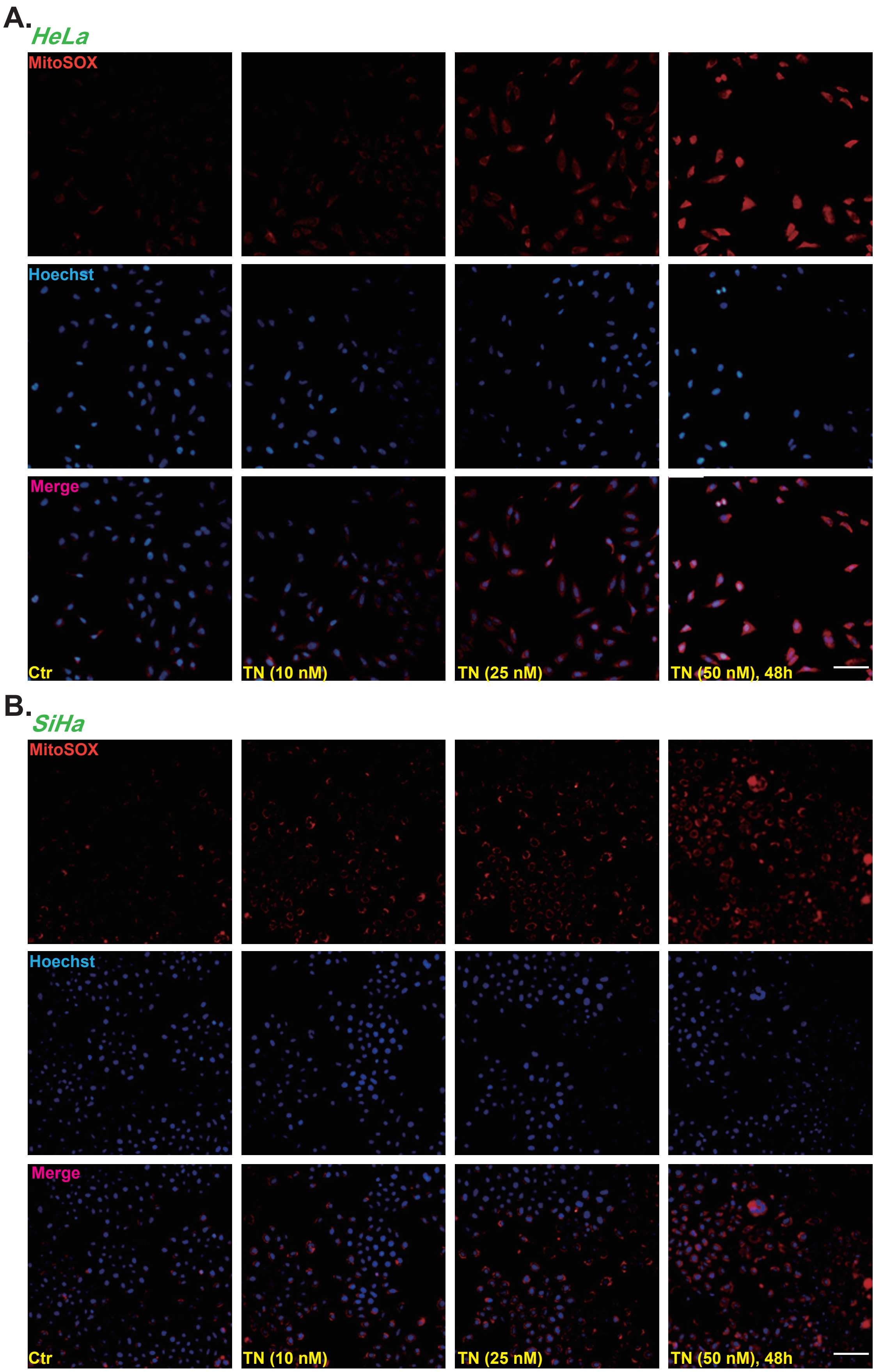

Figure S1: TN induced cervical cancer cell oxidative stress in a dose-dependent manner. Detection of mitochondrial superoxide (red) production by the MitoSOX staining assay in HeLa (A) and SiHa (B) cells. Scale bar = 100μm. n = 3 per group.
